# Association between coeliac disease and cardiovascular disease: prospective analysis of UK Biobank data

**DOI:** 10.1136/bmjmed-2022-000371

**Published:** 2023-01-04

**Authors:** Megan Conroy, Naomi Allen, Ben Lacey, Elizabeth Soilleux, Thomas Littlejohns

**Affiliations:** 1 Nuffield Department of Population Health, University of Oxford, Oxford, UK; 2 UK Biobank, Stockport, UK; 3 Department of Pathology, Unviersity of Cambridge, Cambridge, CB2 1QP

**Keywords:** Epidemiology, Cardiology, Celiac disease

## Abstract

**Objectives:**

To investigate whether people with coeliac disease are at increased risk of cardiovascular disease, including ischaemic heart disease, myocardial infarction, and stroke.

**Design:**

Prospective analysis of a large cohort study.

**Setting:**

UK Biobank database.

**Participants:**

469 095 adults, of which 2083 had coeliac disease, aged 40-69 years from England, Scotland, and Wales between 2006 and 2010 without cardiovascular disease at baseline.

**Main outcome measure:**

A composite primary outcome was relative risk of cardiovascular disease, ischaemic heart disease, myocardial infarction, and stroke in people with coeliac disease compared with people who do not have coeliac disease, assessed using Cox proportional hazard models.

**Results:**

40 687 incident cardiovascular disease events occurred over a median follow-up of 12.4 years (interquartile range 11.5-13.1), with 218 events among people with coeliac disease. Participants with coeliac disease were more likely to have a lower body mass index and systolic blood pressure, less likely to smoke, and more likely to have an ideal cardiovascular risk score than people who do not have coeliac disease. Despite this, participants with coeliac disease had an incidence rate of 9.0 cardiovascular disease cases per 1000 person years (95% confidence interval 7.9 to 10.3) compared with 7.4 per 1000 person years (7.3 to 7.4) in people with no coeliac disease. Coeliac disease was associated with an increased risk of cardiovascular disease (hazard ratio 1.27 (95% confidence interval 1.11 to 1.45)), which was not influenced by adjusting for lifestyle factors (1.27 (1.11 to 1.45)), but was strengthened by further adjusting for other cardiovascular risk factors (1.44 (1.26 to 1.65)). Similar associations were identified for ischaemic heart disease and myocardial infarction but fewer stroke events were reported and no evidence of an association between coeliac disease and risk of stroke.

**Conclusions:**

Individuals with coeliac disease had a lower prevalence of traditional cardiovascular risk factors but had a higher risk of developing cardiovascular disease than did people with no coeliac disease. Cardiovascular risk scores used in clinical practice might therefore not adequately capture the excess risk of cardiovascular disease in people with coeliac disease, and clinicians should be aware of the need to optimise cardiovascular health in this population.

**WHAT IS ALREADY KNOWN ON THIS TOPIC**

- Evidence is conflicting on whether coeliac disease is associated with a higher risk of cardiovascular disease, with previous research being conducted in small cohorts or using registry data sources with limited sociodemographic and lifestyle data
- Previous studies have tended not to take traditional cardiovascular risk factors, such as blood pressure or serum total cholesterol, into account when examining the association between coeliac disease and cardiovascular disease, despite research showing a healthier cardiovascular profile in people with coeliac disease

**WHAT THIS STUDY ADDS**

- Individuals with coeliac disease had a lower prevalence of traditional cardiovascular risk factors, such as systolic blood pressure, total cholesterol, and body mass index, but a higher risk of developing cardiovascular disease, than people with no coeliac disease

**HOW THIS STUDY MIGHT AFFECT RESEARCH, PRACTICE, OR POLICY**

- Cardiovascular risk scores need to adequately account for the elevated risk among people with coeliac disease; people with coeliac disease and their clinicians need to be aware of their higher cardiovascular risk and to take relevant action; and further research is needed to understand the mechanisms underlying these associations

## Introduction

Coeliac disease is an autoimmune disorder resulting in an immune reaction to gluten, a protein found in barley, wheat, and rye. This disease occurs in about 1% of the UK population,^1 2^ although prevalence is increasing, partly due to improved diagnostics.^2^ Coeliac disease is more common in women and is typically diagnosed in childhood and adolescence or at ages 40-60 years, but can occur throughout the life course.^3^ Symptoms include diarrhoea, weight loss, anaemia, chronic fatigue, and other intestinal and extraintestinal symptoms.^3^ Comorbidities are common in people with coeliac disease, with an increased risk of osteoporosis^4^ and some cancers, including non-Hodgkin's lymphoma and small intestinal adenocarcinoma,^5^ as well as anaemias and other autoimmune conditions such as dermatitis herpetiformis.^3 4^ Treatment for coeliac disease involves following a strict gluten-free diet,^3^ which helps to alleviate symptoms and reduces the risk of comorbidities but does not eliminate the condition.^3^

Some studies have reported that coeliac disease is associated with an increased risk of cardiovascular disease, including ischaemic heart disease and stroke.^6–14^ However, evidence is conflicting^15–18^ and is largely based on case-control, cross sectional, or longitudinal studies that do not have complete or detailed information on cardiovascular risk factors and other potentially confounding factors. Based on this conflicting evidence, cardiovascular disease is not currently considered a complication of coeliac disease by National Institute for Health and Care Excellence, the body that sets guidelines for clinical practice in the UK.^19^

Previous studies have tended not to explore the role of traditional cardiovascular disease factors, such as blood pressure or serum total cholesterol, when examining the association between coeliac disease and cardiovascular disease, despite research showing a healthier cardiovascular profile in people with coeliac disease.^7^ We report the findings on the association between coeliac disease and cardiovascular disease using the UK Biobank data,^20^ a large scale, prospective cohort study, and assess whether any association is independent of traditional cardiovascular disease risk factors.

## Methods

### Study design and participants

UK Biobank is a population based cohort study that recruited about 500 000 adults aged 40-69 years from England, Scotland, and Wales between 2006 and 2010.^20^ Participants attended a baseline assessment centre where they provided sociodemographic, lifestyle, and detailed self-reported health information via a touchscreen questionnaire and verbal interview, had physical measurements taken, and provided non-fasting blood samples. A series of serum biomarkers relevant to cardiovascular disease (total cholesterol, triglycerides, glucose, low density lipoprotein, HbA1c, and C reactive protein) were measured in all participants using a Beckman Coulter AU5800 clinical chemistry analyser^21^ and Bio-Rad Variant II Turbo analyser.^22^

All participants provided consent for ongoing linkage to electronic health records to allow longitudinal collection of health events. Hospital inpatient records were obtained from Hospital Episode Statistics for England (available from 1996 until 30 September 2021), Scottish Morbidity Record for Scotland (available from 1981 until 31 July 2021), and Patient Episode Database for Wales (available from 1998 until 28 February 2018). Death records were obtained from NHS Digital (England and Wales; available from the start of recruitment in 2006 until 30 September 2021) and NHS Central Register, National Register of Scotland (Scotland; available from the start of recruitment in 2006 until 31 October 2021).

### Assessment of coeliac disease

Coeliac disease status at recruitment was ascertained by use of a combination of self-reported and hospital inpatient diagnosis. For self-report, participants were asked during the touchscreen questionnaire at baseline: "Has a doctor ever told you that you have had any serious medical conditions or disabilities?" If they answered "yes," then this answer was followed up by a trained nurse during a verbal interview, with coeliac disease one of the medical conditions reported. For the hospital inpatient records, coeliac disease diagnosis was based on International Classification of Diseases (online supplemental table 1) prior to the baseline assessment.

10.1136/bmjmed-2022-000371.supp1 Supplementary data

### Assessment of cardiovascular disease

Incident cardiovascular disease was ascertained from the hospital inpatient and death records. Secondary outcomes were ischaemic heart disease, myocardial infarction, and stroke. A composite cardiovascular disease outcome (ischaemic heart disease, myocardial infarction and stroke combined) was derived as the main outcome (codes available in online supplemental table 1). Any participant self-reporting heart disease or stroke at baseline, or who had cardiovascular disease diagnostic codes in the hospital inpatient data prior to recruitment, was excluded from the analysis (codes available in online supplemental table 1).

### Covariates and cardiovascular risk factors

Socioeconomic status was measured using the Townsend deprivation index score, assigned to each participant using their home postcode at recruitment (grouped into five groups (quintiles)).^23^ The assessment centre location was used to derive region (England (North East, North West, Yorkshire and Humber, East Midlands, West Midlands, South, South West, London), Scotland, Wales). Ethnicity (white, non-white), highest education level (GCSE, A Levels, higher education, none of the above), alcohol consumption (never, special occasions only, one to three times per month, one to two times per week, three to four times per week, daily), smoking status (never, previous, current), family history of heart disease (none, one parent, both parents), medication use (antihypertensive and cholesterol lowering; yes or no) and physical activity were self-reported via the touchscreen questionnaire. The International Physical Activity Questionnaire^24^ guidelines were used to derive low, moderate, and high self-reported physical activity levels. Body mass index was derived from weight (measured by the Tanita BC4 18MA body composition analyser) and standing height measured at recruitment. Systolic and diastolic blood pressure was measured twice, with at least one minute between measurements, using an Omron 705 IT electronic blood pressure monitor with the participant in a seated position. For the prospective analysis, a mean of the two readings was derived and categorised into quintiles. Diabetes was self-reported at the baseline assessment visit or a hospital diagnosis prior to recruitment (codes available in online supplemental table 1). Furthermore, diabetes was categorised into type 1, 2, and unspecified, because the association between coeliac disease and diabetes is type specific (see online supplemental methods and supplemental table 1). For the prospective analysis, the composite definition was used due to small numbers of participants with type 1 diabetes. For the prospective analysis, total cholesterol, blood glucose, systolic blood pressure, triglyceride, low density lipoprotein, C reactive protein, and HbA1c were grouped into fifths. For all variables where data was missing, an additional category for missing data was included. A cardiovascular risk score was generated based on a modified version of the American Heart Association's Life's Simple Seven risk score (LS7).^25 26^ This score was selected because it is well validated,^26 27^ the data required to construct it is readily available in UK Biobank, and has previously been used in UK Biobank research^25^ The score used in these analyses comprise six known cardiovascular risk factors (smoking, physical activity, total cholesterol, diabetes status, blood pressure, body mass index; diet was omitted as the data required to derive fibre and sodium intake were not available) to characterise a person's cardiovascular risk profile as ideal, intermediate or poor (see online supplemental methods and supplemental table 2 for the scoring method).

### Statistical analysis

Participants with prevalent cardiovascular disease (n=33 364) were excluded from the analysis. Of the serum biomarkers of interest (total cholesterol, glucose, HbA1c, triglyceride, low density lipoprotein, and C reactive protein), the distribution of C reactive protein and triglycerides were right skewed and the data were log transformed to reduce departure from normality. Demographic and cardiovascular disease risk factor variables included in the LS7 were cross tabulated by coeliac disease status. Means and proportions were adjusted to the baseline distribution for age, sex, Townsend score, education, and ethnicity to allow better comparison of co-variates between participants with and without coeliac disease.^28^ All variables of interest were examined for missing data. The number of variables with missing data was calculated for each participant, and this number was examined to investigate the proportion of participants missing data on more than two variables. Most (12 of 16) of the variables had some missing data, with this ranging from 0.1% of participants (Townsend Score) to 14.5% (non-fasting glucose) (online supplemental table 3). Fewer than 3.5% of participants had missing data for more than two variables (online supplemental table 4).

Incident rates for cardiovascular disease were calculated and stratified by risk score category (ideal, intermediate, and poor). Cox proportional hazards models were used to calculate the hazard ratio for the cardiovascular disease outcomes of interest. Three models were built: model A was adjusted for standard covariates (adjusted for sex, Townsend score, education, region, year of birth, year of recruitment, and ethnicity); model B was further adjusted for lifestyle factors (as model A plus smoking, alcohol consumption, and physical activity); and model C was further adjusted for cardiovascular risk factors (as model B plus family history of heart disease, total cholesterol, glucose, antihypertensive use, cholesterol lowering medication use, and diabetes). The censoring date was the date of first diagnosis of an outcome of interest (either cardiovascular disease, ischaemic heart disease, stroke, or myocardial infarction), date of death, date of lost to follow-up, or last date of available hospital record, whichever came first. The proportional hazards assumption was examined graphically via log-log plots. The models were stratified for year of birth, year of recruitment, alcohol consumption, and body mass index because these factors violated the proportional hazards assumption. Age was used as the underlying time variable. Where models included covariates with missing data, a separate category of the given covariate was used; multiple imputation of missing data by chained equation, with 35 iterations,^29^ was performed as sensitivity analysis (model D).

We conducted secondary analyses to investigate whether increased inflammation mediated the association between coeliac disease and cardiovascular disease risk by including C reactive protein as a further covariate in the model. Total cholesterol and glucose concentrations were included in the main models to replicate the inclusion in the LS7 risk score. To investigate if the association remained the same with other known cardiovascular biomarkers, total cholesterol and glucose concentrations were replaced with triglyceride, low density lipoprotein, and HbA1c as variables. Participants provided a date of first diagnosis, which was converted to age at diagnosis by UK Biobank. Using this date and the date of recruitment, time since coeliac disease diagnosis was categorised as no coeliac disease, coeliac disease for <10 years, and coeliac disease for ≥10 years at baseline. Models A, B, and C were repeated with time since coeliac disease diagnosis as the exposure and cardiovascular disease as the outcome.

To further investigate the differences in incidence rates of cardiovascular disease by LS7, a joint effects analysis and analyses within groups were undertaken. A joint effects variable was generated that categorised participants by both their coeliac disease status and LS7 category, (no coeliac disease/ideal, no coeliac disease/intermediate, no coeliac disease/poor, coeliac disease/ideal, coeliac disease/intermediate, coeliac disease/poor). Cox proportional hazards modelling was used to investigate the joint effects of coeliac disease status and LS7, adjusted as for model A (as the risk score takes the other potential confounders and effect modifiers into account). For a within group analysis, model A was stratified by LS7 risk score category.

We used Stata SE version 17 (StatCorp, College Station, TX) for our analyses.

### Patient and public involvement

Participants were not involved in the development of the specific research question or outcome measures for this article. Participants were involved in developing the ethics and governance framework for UK Biobank and have been engaged in the progress of UK Biobank through follow-up questionnaires and additional assessment visits. UK Biobank keeps participants informed of all research output through the study website (https://www.ukbiobank.ac.uk/explore-your-participation), participant events, and newsletters.

## Results

Of 502 459 UK Biobank participants, 469 095 were included in this study (33 364 participants had prevalent cardiovascular disease and were excluded from further analyses) and 2083 participants had coeliac disease. During follow-up, 1236 (0.3%) of participants were lost to follow-up (owing to leaving the UK), 1435 (0.3%) were diagnosed with coeliac disease, and 24 707 (5.3%) participants died.

Compared with people who do not have coeliac disease, participants with coeliac disease were more likely to be women (55.8% *v* 71.5%) and of a white ethnic background (94.6% *v* 98.4%) (table 1). Coeliac disease participants also had a lower body mass index, consumed less alcohol, less likely to smoke, more likely to report a family history of heart disease, had lower total cholesterol and C reactive protein concentration, lower mean systolic blood pressure, were less likely to be diagnosed with type 2 diabetes, more likely to be diagnosed with type 1 diabetes and less likely to use cholesterol lowering or antihypertensive medication, when adjusted for age, sex, socioeconomic status, education, and ethnicity (table 2). Compared with those without coeliac disease, those with coeliac disease were more likely to have a so-called ideal cardiovascular risk score (23.3% *v* 14.3%), and were less likely to have a poor risk score (5.0% *v* 8.6%) (table 2). The unadjusted distribution of baseline characteristics were similar to the adjusted results (online supplemental table 5).

**Table 1:** Baseline characteristics of the 469 095 participants included in the main analysis, by pre-existing coeliac disease. Data are number (%), unless otherwise specified

	No coeliac disease (n=467 012)	Coeliac disease (n=2083)
Mean (SD) age (years)	6.7 (8.1)	58.1 (7.9)
Women	260 471 (55.8)	1489 (71.5)
Ethnicity other than white	25 082 (5.4)	33 (1.6)
Wheat-free diet*	6347 (1.4)	1742 (84.0)
Education level		
No qualifications	74 082 (15.9)	404 (19.4)
GCSE	78 135 (16.7)	368 (17.7)
A levels	25 645 (5.5)	105 (5.0)
Higher education	279 977 (60.0)	1184 (56.8)
Townsend deprivation index†		
1 (most affluent)	94 967 (20.3)	461 (22.1)
2	93 818 (20.1)	435 (20.9)
3	93 780 (20.1)	437 (21.0)
4	93 266 (20.0)	374 (18.0)
5 (most deprived)	90 602 (19.4)	374 (18.0)

SD=standard deviation.

*UK Biobank asked participants if they ate a wheat-free diet, so gluten-free diet consumption cannot be ascertained.

†Quintile defined by baseline distribution of UK Biobank population.

**Table 2:** Distribution of lifestyle and cardiovascular risk factors, by pre-existing coeliac disease

	No coeliac disease	Coeliac disease
Body mass index	27.3 (27.3 to 27.3)	25.8 (25.6 to 26.0)
Total cholesterol (mmol/L)	5.8 (5.8 to 5.8)	5.5 (5.4 to 5.5)
Log high sensitivity C reactive protein (mg/L)	0.3 (0.3 to 0.3)	0.1 (0.1 to 0.2)
Non-fasting serum glucose (mmol/L)	5.1 (5.1 to 5.1)	5.1 (5.0 to 5.1)
Low-density lipoprotein (mmol/L)	3.6 (3.6 to 3.6)	3.4 (3.4 to 3.5)
Triglyceride (mmol/L)	1.7 (1.7 to 1.7)	1.5 (1.5 to 1.6)
Glycated haemoglobin (HbA1c) (mmol/mol)	35.9 (35.8 to 35.9)	35.8 (35.5 to 36.0)
Systolic blood pressure (mm Hg)	137.8 (137.8 to 137.9)	135.7 (135.0 to 136.4)
Diastolic blood pressure (mm Hg)	82.4 (82.4 to 82.5)	80.9 (80.4 to 81.3)
Diabetes (%):	4.4 (4.4 to 4.5)	3.2 (2.4 to 4.0)
Type 1 diabetes	0.3 (0.3 to 0.3)	1.0 (0.5 to 1.4)
Type 2 diabetes	3.7 (3.6 to 3.7)	1.8 (1.2 to 2.4)
Current smoker (%)	10.4 (10.3 to 10.5)	7.6 (6.5 to 8.8)
At least weekly alcohol drinker (%)	49.3 (49.1 to 49.4)	47.2 (45.0 to 49.3)
High levels of physical activity (%)*	31.7 (31.5 to 31.8)	29.3 (27.3 to 31.2)
Using blood pressure lowering medication (%)	17.7 (17.6 to 17.8)	13.7 (12.3 to 15.1)
Using cholesterol lowering medication (%)	13.2 (13.1 to 13.3)	8.3 (7.2 to 9.4)
Family history of cardiovascular disease (%)	41.6 (41.5 to 41.8)	44.1 (41.9 to 46.4)
Ideal cardiovascular disease risk score (%)†	14.3 (14.2 to 14.4)	23.3 (21.6 to 25.1)

Data are percentage (95% confidence interval) or mean (95% confidence interval), adjusted for age, sex, Townsend deprivation index score, education, and ethnicity. Numbers of participants and data missing are presented in the supplementary files.

*High physical activity derived according to the International Physical Activity questionnaire.

†Ideal cardiovascular disease risk score defined according to the American Heart Association's Life's Simple Seven risk score.

Over a median follow-up of 12.4 years (interquartile range 11.5-13.1), 40 687 cardiovascular disease events, 33 556 ischaemic heart disease events, 8859 stroke events, and 12 853 myocardial infarction events occurred.

Participants with coeliac disease had a higher absolute incidence of cardiovascular disease compared with people with no coeliac disease (9.03 per 1000 person years (95% confidence interval 7.90 to 10.31) *v* 7.37 (7.30 to 7.44), P for incidence rate difference <0.001).

Coeliac disease was associated with a 27% increased risk of cardiovascular disease compared with participants who did not have coeliac disease (hazard ratio 1.27 (95% confidence interval 1.11 to 1.45)). The results remained similar after further adjustment for lifestyle factors (1.27 (1.11 to 1.45)), whereas adjustment for cardiovascular risk factors increased the strength of the association (1.44 (1.26 to 1.65)) (table 3). The pattern of associations were similar for ischaemic heart disease and myocardial infarction (table 3). Fewer events of stroke occurred than for other endpoints, and no association was noted between coeliac disease and the risk of stroke.

**Table 3:** Association between coeliac disease and cardiovascular disease (CVD) outcomes, with progressive adjustments

	Cases in participants with coeliac disease (n=2083)	Cases in participants with no coeliac disease (n=467 012)	Hazard ratio (95% CI)	P value	χ^2^
All CVD disease:					
Standard adjustments	218	40 469	1.27 (1.11 to 1.45)	<0.001	11.1
Lifestyle factors	218	40 469	1.27 (1.11 to 1.45)	<0.001	11.2
Major CVD risk factors	218	40 469	1.44 (1.26 to 1.65)	<0.001	25.3
Ischaemic heart disease:					
Standard adjustments	182	33 374	1.30 (1.12 to 1.50)	<0.001	11.3
Lifestyle factors	182	33 374	1.30 (1.12 to 1.50)	<0.001	11.2
Major CVD risk factors	182	33 374	1.5 (1.30 to 1.75)	<0.001	26.5
Myocardial Infarction:					
Standard adjustments	70	12 783	1.34 (1.06 to 1.70)	0.01	5.5
Lifestyle factors	70	12 783	1.35 (1.07 to 1.71)	0.01	5.8
Major CVD risk factors	70	12 783	1.59 (1.25 to 2.01)	<0.001	12.7
Stroke:					
Standard adjustments	46	8813	1.13 (0.85 to 1.52)	0.39	0.7
Lifestyle factor	46	8813	1.15 (0.86 to 1.53)	0.35	0.8
Major CVD risk factors	46	8813	1.20 (0.89 to 1.60)	0.23	1.4

Hazard ratios are progressively adjusted for a standard set of adjustments, lifestyle factors, and major CVD risk factors. Standard adjustments are for region, sex, Townsend deprivation index score, education, year of birth, year of recruitment and ethnicity, with age as underlying time variable; lifestyle factors adjustments are for physical activity, smoking, and alcohol consumption; and major cardiovascular risk factors adjustments are for body mass index, total cholesterol, glucose, blood pressure, antihypertensive medication, cholesterol lowering medication, family history of heart disease, and diabetes. CI=confidence interval.

The findings remained similar after using multiple imputation to account for missing data (model D online supplemental table 6). In a secondary analysis that aimed to investigate whether increased inflammation could explain the increased risk of cardiovascular disease, ischaemic heart disease, or myocardial infarction, all models were further adjusted for C reactive protein. No attenuation of the results was identified (model C plus log C reactive protein: hazard ratio for cardiovascular disease 1.39 (95% confidence interval 1.21 to 1.60), P<0.001; ischaemic heart disease 1.49 (1.28 to 1.74), P<0.001; and myocardial infarction 1.60 (1.25 to 2.05), P<0.001). No difference was noted in the results when triglyceride, low density lipoprotein, and HbA1c concentrations were included rather than total cholesterol and glucose concentrations (cardiovascular disease 1.39 (1.21 to 1.60), P<0.001; ischaemic heart disease 1.51 (1.30 to 1.76), P<0.001; myocardial infarction 1.66 (1.31 to 2.10), P<0.001; and stroke 1.09 (0.80 to 1.50), P=0.58).

Evidence of a dose-response association between time since coeliac disease diagnosis and risk of cardiovascular disease was reported. Compared with people who do not have coeliac disease, people who had coeliac disease for less than 10 years had a 30% increased risk (model C 1.30 (95% confidence interval 1.07 to 1.57)), and people who had coeliac disease for 10 years or more had a 34% increased risk (1.34 (1.11 to 1.61), P value for trend<0.001; online supplemental table 7).

**Figure 1:**
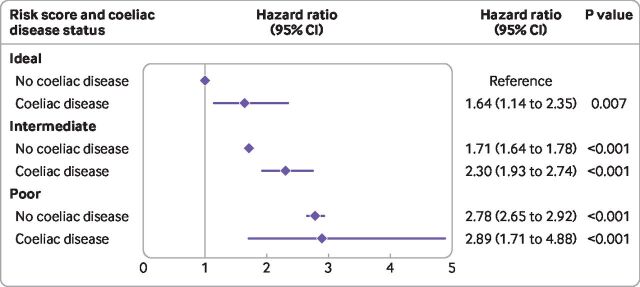
Risk of incident cardiovascular disease by coeliac disease and cardiovascular risk score. Hazard ratios adjusted for region, sex, Townsend score, education, year of birth, year of recruitment, and ethnicity, with age as underlying time variable. The vertical line represents hazard ratio of 1. Risk score was defined using the American Heart Association's Life's Simple Seven score. CI=confidence interval

When investigating the potential joint effects of coeliac disease and cardiovascular risk score and incident cardiovascular disease, people with coeliac disease and an ideal risk score had more than 60% increased risk (hazard ratio 1.64 (95% confidence interval 1.14 to 2.35) P=0.007; figure 1) compared with people with no coeliac disease and an ideal risk score of cardiovascular disease. Participants with an intermediate risk score and coeliac disease had 2.3 times an increased risk (2.30 (1.93 to 2.74), P<0.001) and people with a poor risk score and coeliac disease had almost three times an increased risk (2.89 (1.71 to 4.88), P<0.001), compared with those with an ideal score and no coeliac disease. The increased risk noted with a poor risk score compared with ideal risk was similar in people with coeliac disease (2.89 (1.17 to 4.88)) and in people who do not have coeliac disease (2.78 (2.65 to 2.92)), indicating that coeliac disease did not further amplify the risk of cardiovascular disease in people who are already at high risk of cardiovascular disease (figure 1), but the number of participants in this group was small (table 4). As such, within the ideal and intermediate risk score categories, the hazard ratios for incident cardiovascular disease were 1.57 ((95% confidence interval 1.10 to 2.35), P=0.01) for those with coeliac disease compared with 1.35 ((1.14 to 1.60), P<0.001) for those who do not have coeliac disease (table 4); however, no association was noted for for people in the poor risk score category (1.03 (0.61 to 1.74)).

**Table 4:** Analysis within groups for association between coeliac disease and cardiovascular disease, within risk score category

Risk score category and cardiovascular disease status	Time at risk (years)	No of participants	Cases	Hazard ratio (95% CI)	P value
Ideal risk score:					
No cardiovascular disease	811 853	66 840	2370	1 (ref)	—
Cardiovascular disease	5710	479	30	1.57 (1.09 to 2.25)	0.01
Intermediate risk score:					
No cardiovascular disease	3 427 237	291 199	24 275	1 (ref)	—
Cardiovascular disease	13 972	1204	135	1.35 (1.14 to 1.60)	<0.001
Poor risk score:					
No cardiovascular disease	454 795	40 207	5582	1 (ref)	—
Cardiovascular disease	1107	103	14	1.03 (0.61 to 1.74)	0.92

Adjusted for region, sex, Townsend score, education, year of birth, year of recruitment and ethnicity, age as underlying time variable. CI=confidence interval.

## Discussion

### Principal findings

To our knowledge, this study is the largest longitudinal study to investigate the association between coeliac disease and cardiovascular disease that allowed for robust adjustment of potential confounders. We adjusted for including a wide range of lifestyle, medical, and cardiovascular risk factors (including biomarkers), showing that the increased risk is not explained by traditional cardiovascular risk factors. Furthermore, we were able to investigate dose-response by time since coeliac disease diagnosis. We found the risk of cardiovascular disease increased as time since coeliac disease increased, suggesting that coeliac disease might increase the risk of cardiovascular disease, with the longer the exposure, the higher the risk. Use of self-reported health data and hospital inpatient data from the mid-1990s meant most cases of coeliac disease are likely to have been captured over time.

### Comparison with other studies

Our findings complement previous research that reported that specific cardiovascular disease conditions were over-represented in individuals with coeliac disease,^11 13 14^ including a UK Biobank study that investigated across all diseases in a hypothesis-free approach.^12^ We build on these findings by accounting for the role of cardiovascular health in the analysis. Previous research investigating coeliac disease with risk of cardiovascular disease has produced conflicting findings, showing either an increased risk^6–14^ or no association.^15–18^ However, studies vary in adjustment, with some studies having minimal adjustments (eg, for age, sex, and socioeconomic status)^6 12 15 16^ and others further adjusting for comorbidities (such as other autoimmune diseases and hypertension).^8 10 11 13 18^ Two meta-analyses have been undertaken, one identifying an increased risk of incident stroke,^9^ and the other identifying an increased risk of death from stroke.^17^ Neither identified an association between coeliac disease and incident cardiovascular disease, myocardial infarction, or cardiovascular disease death.^11 18^ In both meta-analyses, the included studies were of low quality, with little adjustment for lifestyle and health related confounders. The authors of both meta-analyses note that large prospective studies are needed, which adequately adjust for confounding, to investigate the association between coeliac disease and cardiovascular disease. The absence of association with stroke and coeliac disease identified in this study could be due to a lack of power because the number of stroke events in the coeliac disease participants was low. To our knowledge, no previous study has taken into account cardiovascular risk score or biochemical risk factors for cardiovascular disease, such as cholesterol, although patients with coeliac disease have a better cardiovascular risk profile than do people who do not have coeliac disease.^7^ Our research has highlighted that the increased risk of cardiovascular disease is more marked in those with an ideal cardiovascular risk profile.

Numerous autoimmune diseases have been found to be associated with an increased risk of cardiovascular disease.^14^ One hypothesis for the increased risk is that increased systemic inflammation associated with coeliac disease could subsequently increase the risk of cardiovascular disease.^30–32^ In our study, the results remained similar when adjusting for C reactive protein, a marker of systemic inflammation. C reactive protein might be a poor marker for inflammation in patients with coeliac disease,^7 33^ as found in other auto-immune diseases,^34^ although small case control studies have identified an increased C reactive protein in patients with coeliac disease.^32 35^ Systemic inflammation is known to trigger atherosclerosis,^36^ and previous studies have found that patients with coeliac disease have increased intima-medial thickness, reduced elasticity of the ascending aorta, and endothelial dysfunction.^35 37 38^ A similar association with increased risk of atherosclerosis has been identified in other auto-immune disorders, such as rheumatoid arthritis and systemic lupus erythematosus.^39 40^ Some studies have shown that a gluten-free diet reduces inflammation and cardiovascular disease in patients with coeliac disease.^6 41^ Our study cannot investigate whether adherence to a gluten-free diet reduced inflammation and, therefore, whether this factor affected the risk of cardiovascular disease in patients with coeliac disease.

A further hypothesis is that the consumption of a gluten-free diet might increase the risk of cardiovascular disease because gluten-free foods are higher in saturated fats, sugar, and salt,^42^ or because a gluten-free diet limits consumption of complex whole grains.^43^ Previous studies have identified changes in cardiovascular biomarkers (such as body mass index, total cholesterol, and triglycerides) after implementing a gluten-free diet, but these changes did not indicate to a better or worse cardiovascular profile.^44^ We were unable to explore the direct affect of diet (due to only having data for a wheat-free diet, and the small proportion of participants with coeliac disease reporting not eating a wheat-free diet). However, any downstream effect of diet is unlikely to be reflected in the cardiovascular risk factors we investigated in the current study; a Cochrane review found no association between gluten-free diet and cardiovascular disease risk.^45^ Another potential mechanism for the increased risk noted is through micronutrient deficiencies. Micronutrient deficiencies (such as vitamins A, B, D, and E) have been associated with cardiovascular disease outcomes, although evidence is conflicting.^46^ Micronutrient deficiencies can occur in patients with coeliac disease who do not adhere to a gluten-free diet (due to malabsorption from villi atrophy), but also occurs in people who do adhere to a gluten-free diet, due to the inadequate micronutrients in a gluten-free diet.^47^ However, micronutrient deficiencies are common in the UK population (especially among women),^48 49^ and so is unlikely to fully explain the increased risk of cardiovascular disease seen in coeliac disease participants identified in this study.

### Strengths and limitations

The study was limited in that the traditional cardiovascular disease risk factors (including biomarkers and blood pressure) were only measured in the full sample at recruitment, and hence, we could not investigate the impact of changes in cardiovascular disease risk factors over time on risk. Coeliac disease status was ascertained by use of a combination of self-reported and hospital inpatient data. This process might have led to an under ascertainment of cases due to participants not self-reporting a diagnosis and not having a diagnosis recorded in their hospital record (because of how diseases are recorded in Hospital Episode Statistics). As a result of the amount of missingness for some variables (ie, physical activity and some biomarker data), missing data were maintained in the models to ensure adequate power. Although the inclusion of missing data has the potential to bias the results, these variables were covariates and fewer than 3.5% of participants had missing data for more than two variables. Furthermore, multiple imputation showed no change in results when accounting for the missing data, suggesting no bias was introduced by use of this method. A gluten-free diet was not taken into account as the diet questionnaire only asked whether a wheat-free diet was followed (gluten is identified in other grains, such as barley and rye) and the number of participants with coeliac disease who reported not following a wheat-free diet was small, so the impact of a gluten-free diet could not be assessed. The association could be due to ascertainment bias because people with a chronic disease, such a coeliac disease, are more likely to have a hospital record, and, as such, are also more likely to have a cardiovascular disease recorded in their hospital record.^50 51^ However, analyses that were restricted to myocardial infarction as the outcome (which is predominantly diagnosed in hospital) were very similar to those for cardiovascular disease as a whole, suggesting that ascertainment bias is unlikely to have influenced the results in any meaningful way.

Although this study is large, it lacked power to assess specific cardiovascular disease subtypes and so joint effects of cardiovascular risk score and coeliac disease status on cardiovascular disease subtypes was not possible. Possible conclusions were also not made for the lack of difference in risk for people with a poor cardiovascular risk score (which could reflect a lack of power in this subgroup or could indicate that the cardio-protective therapies that are likely implemented for those with a poor risk score are protective against cardiovascular disease in those with coeliac disease). As with all observational studies, residual confounding likely remains and causality cannot be determined. As UK Biobank is a volunteer based cohort, the so-called healthy volunteer effect might affect consequent findings,^52^ which could explain the lower prevalence of coeliac disease noted in this study compared with the general population. Additionally, the absolute incidence rates reported in this paper are unlikely to be generalisable to the wider population.^53^ Nevertheless, the internal associations identified are likely to be generalisable to the population as a whole owing to the wide heterogeneity of risk factors studied.^53^

### Conclusion

This study highlights the importance of cardiovascular disease as a potential complication of coeliac disease. Further research into the drivers and mechanistic pathways of this association is warranted. In addition, an investigation is warranted into the extent to which any risk reduction is reported by adherence to a gluten-free diet in people with coeliac disease, or whether a gluten-free diet itself contributes to the increased risk identified. Furthermore, consideration should be given to inclusion of coeliac disease as a risk factor in cardiovascular disease risk prediction models, such as the QRISK model,^54^ which currently includes other autoimmune conditions (systemic lupus erythematosus and rheumatoid arthritis) as risk factors. Given the increased rates of cardiovascular disease reported in people with coeliac disease who have an ideal and moderate cardiovascular disease risk score, clinicians should make patients with coeliac disease aware of their elevated risk, and work with their patients to optimise their cardiovascular health.

## Data Availability

Data are available upon reasonable request. UK Biobank is an open access resource. Bona fide researchers can apply to use the UK Biobank dataset by registering and applying at http://ukbiobank.ac.uk/register-apply/ http://ukbiobank.ac.uk/register-apply/. All results presented in this manuscript, including the code used to generate them, will be returned to UK Biobank within 6 months of publication at which point they are made available for researchers to request (subject to UK Biobank approval).
