# Supplementary material for: Association between coeliac disease and cardiovascular disease: prospective analysis of UK Biobank data
Source: BMJ Med. 2023 Jan 4;2(1):e000371. doi: 10.1136/bmjmed-2022-000371 (PMC9951384; doi:10.1136/bmjmed-2022-000371)
Supplement: Supplementary data [file bmjmed-2022-000371supp001.pdf]

Supplementary Material

Contents

Supplementary Material 1
Supplementary methods 2
Calculation of sub-types of Diabetes Mellitus 2
Calculation of the modified American Heart Association's Life's Simple Seven risk score 2
Supplementary Results 4
Supplementary Table 1: Codes for disease ascertainment 4
Supplementary Table 2: Modified Life's Simple Seven Risk Score 4
Supplementary Table 3: Proportion of variables missing data in co-variates included in fully adjusted model (*model c)* 5
Supplementary Table 4: Proportion of participants with missing data, by number of variables missing data for 5
Supplementary Table 5: Unadjusted and adjusted categorical lifestyle and cardiovascular risk factors distributions. 6
Supplementary Table 6: Association between Coeliac disease and Cardiovascular diseases – multiple imputation results 7
Supplementary Table 7: Association between time since coeliac disease diagnosis and cardiovascular disease risk 7
References 8

Conroy M, *et al. BMJMED* 2023; 2:e000371. doi: 10.1136/bmjmed-2022-000371

Supplementary methods

Calculation of sub-types of Diabetes Mellitus

The sub characterisation of Diabetes Mellitus in UKB is complex[1]. Whilst participants could self-report diabetes type, they could also only state "diabetes". Additionally, whilst ICD10 coding has type 1 and type 2 codes available, there are a number of unspecified codes associated with diabetes. ICD9 coding does not have type 1 and type 2 coding available, rather it has juvenile onset and adult onset, again with a number of unspecified diabetes codes. Previous authors have highlighted how difficult it is to accurately phenotype diabetes subtypes in UKB, although self-reported diabetes status, in association with medication use, seems to be well corroborated by primary-care records[1]. To ascertain diabetes sub-type in this study, the following rules were applied:

Type 1: Participants who either:

a) Self-reported Type 1 Diabetes at baseline;
b) Self-reported Diabetes at baseline and reported starting insulin within 1 year of diagnosis (DF 2986);
c) Had a ICD code associated with Type 1 Diabetes (juvenile onset in ICD9) in their HES record prior to recruitment.

Type 2: Participants who either:

a) Self-reported Type 2 Diabetes at baseline;
b) Self-reported Diabetes at baseline and reported not starting insulin within 1 year of diagnosis (DF 2986);
c) Had a ICD code associated with Type 2 Diabetes (adult onset in ICD9) in their HES record prior to recruitment.

Unspecified: Participants who either:

a) Self-reported Diabetes at baseline and did not answer DF 2986;
b) Had a ICD code associated with unspecified Diabetes in their HES record prior to recruitment
c) Did not have a code associated with either Type 1 or Type 2 diabetes in their HES record prior to recruitment.

Full code lists for each subtype are available in supplementary table 1.

Calculation of the modified American Heart Association's Life's Simple Seven risk score

The American Heart Association's Life's Simple Seven risk score was developed by the American Heart Association as a tool to support the improvement of cardiovascular health[2,3]. It consists of seven modifiable behaviours and health markers; smoking, body mass index (BMI), physical activity, diet, serum total cholesterol, fasting blood glucose and blood pressure. This score has been modified

to be used in UKB data[4]. The dietary data collected at recruitment was not sufficient to calculate a diet score, and so this component was not included. Blood samples collected were not fasting blood samples, so three separate scores were generated; the first used diabetes as a proxy for fasting serum glucose[4], the second used non-fasted serum glucose (>7mmol/l=0; 5.6-7mmol/l and no insulin use OR <5.6mmol/l with insulin use=1; <5.6mmol/l=2)[2] and the third used HbA1c as a proxy for fasting glucose (≥48mmol/mol=0; 42-47.9mmol/mol and no insulin use OR <42mmol/l with insulin use=1; <42mmol/mol=2)[5]. A cox proportional hazards model was run for each cardiovascular risk score and CVD as the outcome. Models were adjusted for year of birth, year of recruit, region, sex, townsend score, ethnicity and education. Age was the underlying time variable. Hazard ratios for CVD for each model were compared, as were the model Akaike information criterion (AIC)[6]. No difference was seen between the hazard ratios for CVD between the three risk scores. AIC and BIC were similar for risk scores 1 and 3, and lower for risk score 2. The cardiovascular risk score using diabetes as a proxy for fasting serum glucose was selected (risk score 1), as this had the least missingness.

Supplementary Results

Supplementary Table 1: Codes for disease ascertainment

Disease	Self-reported Code	ICD9	ICD10
**Coeliac Disease**	1456	5790	K900
**Ischaemic Heart disease**	1074, 1075	410 - 414	I20 – I25
**MI**	1075	410-412	I21-I23, I241, I252
**Stroke**	1081, 1086, 1583	430,431,434,436	I60,I61,I63, I64
**Diabetes**	1220, 1222, 1223	250	E10, E11, E13, E14
**Type 1 Diabetes**	1222	25001, 25011	E10
**Type 2 Diabetes**	1223	25000, 25010	E11
**Unspecified Diabetes**	1220	All other 250 codes	E13, E14

Supplementary Table 2: Modified Life's Simple Seven Risk Score

Risk Factor	Simple 7 score		
	0	1	2
**Smoking**	Current Smoker	Former ≤12months since stopping	Never or stopped ≥12 months prior
**Physical Activity**	None	1-149min/week moderate or 1-74min/week vigorous or 1-149min/week moderate+ vigorous	≥150min/ week moderate or ≥ 75min/ week vigorous or ≥150min/ week moderate+ vigorous
**Total Cholesterol**	>6.21mmol/l	5.172-6.21mmol/l with no cholesterol lowering medication or <5.127mmol/l with cholesterol lowering medication	<1.572mmol/l with no cholesterol lowering medication
**Diabetes**	Self-reported Diabetes and insulin medication	Self-reported diabetes and no insulin medication	No self-reported diabetes and no insulin medication
**Blood Pressure**	SBP ≥140mmHg or DBP ≥90mmHg	SBP 120-139mmHg with no blood pressure lowering medication or DBP 80-89mmHg with no blood pressure lowering medication or <120mmHg SBP/ <80mmHg DBP and blood pressure lowering medication	<120mmHg SBP/ <80mmHg DBP with no blood pressure lowering medication
**BMI**	≥30 kg/m^2	25-<30 kg/m^2	<25 kg/m^2

Sum of scores:
0-5: Poor cardiovascular risk score
6-9: Intermediate cardiovascular risk score
10-12: Ideal cardiovascular risk score

Supplementary Table 3: Proportion of variables missing data in co-variates included in fully adjusted model (*model c)*

Variable	Missing	%	Not missing	%
**Townsend Score**	581	0.1	468,514	99.9
**Sex**	0	0.0	469,095	100.0
**Ethnicity**	2,555	0.5	466,540	99.5
**Smoking**	2,625	0.6	466,470	99.4
**Education**	9,195	2.0	459,900	98.0
**Physical Activity**	107,812	23.0	361,283	77.0
**Family History of Heart Disease**	50,877	10.8	418,218	89.2
**Total Cholesterol**	30,532	6.5	438,563	93.5
**Non-fasting glucose**	67,942	14.5	401,153	85.5
**Systolic Blood Pressure**	1,216	0.3	467,879	99.7
**Antihypertensive use**	0	0.0	469,095	100.0
**Statin use**	7,861	1.7	461,234	98.3
**Prevalent Diabetes**	0	0.0	469,095	100.0
**BMI**	2,707	0.6	466,388	99.4
**Alcohol**	1,366	0.3	467,729	99.7
**Region**	0	0	469,096	100
**Year of Birth**	0	0	469,096	100
**Year of Recruit**	0	0	469,096	100

Supplementary Table 4: Proportion of participants with missing data, by number of variables missing data for

Number of variables missing data	N	%
**0**	276,751	59
**1**	131,749	28.09
**2**	43,737	9.32
**3**	10,179	2.17
**4**	1,834	0.39
**5**	3,348	0.71
**6**	542	0.12
**7**	481	0.1
**8**	70	0.01
**9**	82	0.02
**10**	28	0.01
**11**	292	0.06
**12**	2	0

Supplementary Table 5: Unadjusted and adjusted categorical lifestyle and cardiovascular risk factors distributions.

	Unadjusted*		Adjusted†	
	No Coeliac	Coeliac	No Coeliac	Coeliac
**Body Mass Index**	27.3 (4.8)	25.7 (4.5)	27.3 (27.3-27.3)	25.8 (25.6-26.0)
**<25**	157882 (33.8%)	1024 (49.2%)	33.8% (33.7%-33.9%)	47.1% (45.0%-49.2%)
**25-<30**	197128 (42.2%)	767 (36.8%)	42.2% (42.1%-42.3%)	38.6% (36.5%-40.7%)
**>=30**	109299 (23.4%)	288 (13.8%)	23.4% (23.3%-23.5%)	14.0% (12.5%-15.5%)
**Smoking status**				
**Never**	259248 (55.5%)	1280(61.4%)	55.5% (55.4%-55.7%)	61.2% (59.1%-63.2%)
**Previous**	156679 (33.5%)	656(31.5%)	33.6% (33.4%-33.7%)	30.9% (29.0%-32.9%)
**Current**	48463 (10.4%)	144(6.9%)	10.4% (10.3%-10.5%)	7.6% (6.5%-8.8%)
**Alcohol Consumption**				
**Never**	36068 (7.7%)	202(9.7%)	7.7% (7.6%-7.8%)	9.9% (8.7%-11.1%)
**Special occasions only**	52973 (11.3%)	303(14.5%)	11.3% (11.3%-11.4%)	13.4% (12.0%-14.8%)
**1-3 times per month**	52134 (11.2%)	255(12.2%)	11.2% (11.1%- 1.3%)	11.8% (10.4%-13.1%)
**1-2 times per week**	120807 (25.9%)	526(25.3%)	25.9% (25.7%-26.0%)	25.2% (23.4%-27.1%)
**3-4 times per week**	108602 (23.3%)	442(21.2%)	23.3% (23.1%-23.4%)	21.8% (20.0%-23.6%)
**Daily or almost daily**	95063 (20.4%)	354(17.0%)	20.4% (20.2%-20.5%)	17.6% (16.0%-19.3%)
**Physical Activity‡**				
**Low**	65324 (14.0%)	302(14.5%)	14.0% (13.9%-14.1%)	15.1% (13.5%-16.6%)
**Moderate**	146445 (31.4%)	657(31.5%)	31.4% (31.2%-31.5%)	31.4% (29.5%-33.4%)
**High**	147962 (31.7%)	593(28.5%)	31.7% (31.5%-31.8%)	29.3% (27.3%-31.2%)
**Family History of Heart Disease**				
**No**	243115 (52.1%)	1019(48.9%)	52.1% (51.9%-52.2%)	50.1% (48.0%-52.3%)
**Yes**	173218 (37.1%)	866(41.6%)	37.1% (37.0%-37.2%)	39.6% (37.6%-41.7%)
**Antihypertensive use**				
**No**	376712 (80.7%)	1759 (84.4%)	82.3% (82.2%- 2.4%)	86.3% (84.9%-87.7%)
**Yes**	82464 (17.7%)	299 (14.4%)	17.7% (17.6%-17.8%)	13.7% (12.3%-15.1%)
**Statin use**				
**No**	397556 (85.1%)	1879 (90.2%)	85.1% (85.0%-85.2%)	89.9% (88.7%-91.1%)
**Yes**	61620 (13.2%)	179 (8.6%)	13.2% (13.1%- 3.3%)	8.3% (7.2%-9.4%)
**Diabetes**				
**No**	446312 (95.6%)	2023 (97.1%)	95.6% (95.5%-95.6%)	96.8% (96.0%-97.6%)
**Yes**	20700 (4.4%)	60 (2.9%)	4.4% (4.4%-4.5%)	3.2% (2.4%-4.0%)
**Type 1**	1,511 (0.3%)	17 (0.8%)	0.3% (0.3% – 0.3%)	1.0% (0.5% - 1.4%)
**Type 2**	17,168 (3.7%)	35 (1.7%)	3.7% (3.6% - 3.7%)	1.8% (1.2% - 2.4%)
**Unspecified**	2,153 (0.5%)	6 (0.3%)	0.5% (0.4% - 0.5%)	0.3% (0.1% - 0.5%)
**Cardiovascular risk score§**				
**Poor**	40207 (8.6%)	103 (4.9%)	8.6% (8.5%-8.7%)	5.0% (4.1%-6.0%)
**Intermediate**	291199 (62.4%)	1204 (57.8%)	62.4% (62.2%-62.5%)	57.9% (55.9%-60.0%)
**Ideal**	66840 (14.3%)	479 (23.0%)	14.3% (14.2%-14.4%)	23.3% (21.6%-25.1%)
**Total cholesterol (mmol/L)**	5.8 (1.1)	5.5 (1.1)	5.8 (5.8-5.8)	5.5 (5.4-5.5)
**C-reactive Protein (mg/L)¶**	0.3 (1.1)	0.2 (1.1)	0.3 (0.3-0.3)	0.1 (0.1-0.2)
**Non-fasting glucose (mmol/L)**	5.1 (1.2)	5.0 (1.1)	5.1 (5.1-5.1)	5.1 (5.0-5.1)
**Systolic blood pressure (mmHg)**	137.8 (18.7)	135.9 (19.6)	137.8 (137.8- 37.9)	135.7 (135.0-136.4)
**Diastolic Blood Pressure (mmHg**	82.4 (10.1)	80.4 (10.2)	82.4 (82.4-82.5)	80.9 (80.4-81.3)
**Low-density lipoprotein (mmol/L)**	3.6 (0.9)	3.5 (0.8)	3.6 (3.6-3.6)	3.4 (3.4-3.5)
**Triglycerides (mmol/L) ¶**	1.7 (1.0)	1.5 (0.9)	1.7 (1.7-1.7)	1.5 (1.5-1.6)
**HbA1c (mmol/mol)**	35.9 (6.4)	35.8 (6.1)	35.9 (35.8-35.9)	35.8 (35.5-36.0)

*mean (SD) or N (%) †mean (95% Confidence Interval) or % (95% Confidence Interval). Adjusted for age, sex, townsend score, education and ethnicity. ‡Physical activity defined using the International Physical Activity Questionnaire § Cardiovascular risk score defined using the American Heart Association's Life's Simple 7 risk score ¶ Log transformed to correct for departure from normality

Supplementary Table 6: Association between Coeliac disease and Cardiovascular diseases – multiple imputation results

		Hazard Ratio	95% Confidence Interval	p-value
**Cardiovascular disease**	Model *d*	1.52	1.32 – 1.75	<0.001
**Ischaemic Heart Disease**	Model *d*	1.60	1.37 – 1.87	<0.001
**Stroke**	Model *d*	1.26	0.92 – 1.71	0.15
**Myocardial Infarction**	Model *d*	1.65	1.28 – 2.12	<0.001

Adjusted for region, sex, townsend score, education, year of birth, year of recruitment and ethnicity, physical activity, smoking and alcohol consumption, BMI, total cholesterol, glucose, blood pressure, antihypertensive medication, cholesterol lowering medication, family history of heart disease and diabetes; age as underlying time variable

Supplementary Table 7: Association between time since coeliac disease diagnosis and cardiovascular disease risk

Model	Time since CD onset	No CVD	CVD	HR	95% CI	p-value	p-value for trend	Chi2
**a**	No CD	426543	40469	Reference			<0.001	11.98
	CD < 10 years	968	108	1.22	1.01 - 1.47	0.04		
	Cd >= 10 years	886	110	1.33	1.10 - 1.60	0.003		
**b**	No CD	426543	40469	Reference			<0.001	12.07
	CD < 10 years	968	108	1.22	1.01 - 1.48	0.04		
	Cd >= 10 years	886	110	1.33	1.10 - 1.60	0.003		
**c**	No CD	426543	40469	Reference			<0.001	-3843.24
	CD < 10 years	968	108	1.30	1.07 – 1.57	0.007		
	Cd >= 10 years	886	110	1.34	1.11 – 1.61	0.003		

[1]Model *a*: Minimally adjusted (region, sex, Townsend score, education, year of birth, year of recruitment and ethnicity, age as underlying time variable)

[2]Model *b*: Adjusted for lifestyle factors (as *a* plus physical activity, smoking and alcohol consumption, age as underlying time variable)

[3]Model *c*: Adjusted for cardiovascular risk factors (as model *b* plus BMI, total cholesterol, glucose, blood pressure, antihypertensive medication, cholesterol lowering medication, family history of heart disease and diabetes, age as underlying time variable)

CD: Coeliac disease; HR: Hazard Ratio; CI: Confidence interval

References

1. Eastwood S v, Mathur R, Atkinson M, et al. Algorithms for the Capture and Adjudication of Prevalent and Incident Diabetes in UK Biobank. *PLoS One*. 2016;11(9):e0162388. doi:10.1371/journal.pone.0162388

2. Lloyd-Jones DM, Hong Y, Labarthe D, et al. Defining and setting national goals for cardiovascular health promotion and disease reduction: The american heart association's strategic impact goal through 2020 and beyond. *Circulation*. 2010;121(4):586-613. doi:10.1161/CIRCULATIONAHA.109.192703

3. Garg PK, O'Neal WT, Chen LY, et al. American Heart Association's Life Simple 7 and risk of atrial fibrillation in a population without known cardiovascular disease: The ARIC (Atherosclerosis risk in communities) study. *J Am Heart Assoc*. 2018;7(8). doi:10.1161/JAHA.117.008424

4. Atkins JL, Delgado J, Pilling LC, et al. Impact of Low Cardiovascular Risk Profiles on Geriatric Outcomes: Evidence From 421,000 Participants in Two Cohorts. *The Journals of Gerontology: Series A*. Published online May 21, 2018. doi:10.1093/gerona/gly083

5. Welsh C, Welsh P, Celis-Morales CA, et al. Glycated Hemoglobin, Prediabetes, and the Links to Cardiovascular Disease: Data From UK Biobank. *Diabetes Care*. 2020;43(2):440-445. doi:10.2337/dc19-1683

6. Akaike H. A New Look at the Statistical Model Identification. *IEEE Trans Automat Contr*. 1974;19(6):716-723. doi:10.1109/TAC.1974.1100705
